# Intra-articular Osteoid Osteoma of the Femoral Neck Mimicking Inflammatory Hip Disease: A Case Report and Review of the Literature

**DOI:** 10.7759/cureus.115006

**Published:** 2026-08-22

**Authors:** El Assad Houda, Hamza Toufik, Abderrahim Majjad, Ahmed Bezza

**Affiliations:** 1 Rheumatology, Hôpital Militaire d'Instruction Mohammed V, Rabat, MAR

**Keywords:** case-based review, computed tomography, femoral neck, hip pain, osteoid osteoma

## Abstract

Osteoid osteoma is a benign osteogenic tumor that predominantly affects the long bones of young adults. Intra-articular involvement of the femoral neck is uncommon and may present with atypical clinical features, often leading to a delayed diagnosis. We report the case of a 30-year-old woman presenting with chronic right hip pain of two years’ duration, characterized by nocturnal exacerbation and partial relief with nonsteroidal anti-inflammatory drugs (NSAIDs). Initial evaluation raised suspicion of inflammatory hip disease; however, laboratory investigations were unremarkable. Subsequent CT revealed a well-defined 10 × 8 mm nidus in the inferomedial subcapital region of the right femoral neck, establishing the diagnosis of intra-articular osteoid osteoma. The patient underwent CT-guided percutaneous excision with complete pain relief, and histopathological examination confirmed the diagnosis.

Intra-articular osteoid osteoma of the femoral neck represents a particular diagnostic challenge because its clinical presentation may be atypical, and inflammatory joint disease may initially be considered. MRI findings in intra-articular osteoid osteoma can be nonspecific, as reactive synovitis, joint effusion, and bone marrow edema may obscure the nidus, as reported in the literature. CT remains particularly useful for identifying the nidus when the diagnosis is uncertain. To place this case in context, we performed a narrative review of previously published reports on intra-articular and femoral neck osteoid osteoma, focusing on the clinical presentation, diagnostic pitfalls, imaging findings, and management strategies.

## Introduction

Osteoid osteoma is a benign osteogenic tumor that accounts for approximately 10% of all benign bone tumors and 2%-3% of primary bone tumors. It predominantly affects adolescents and young adults, with the femur and tibia representing the most frequent sites of involvement [1-3]. The hallmark clinical presentation consists of localized pain that is typically worse at night and characteristically relieved by nonsteroidal anti-inflammatory drugs (NSAIDs), a feature attributed to the high prostaglandin production within the tumor nidus [1,3,4].

Intra-articular osteoid osteoma is uncommon, representing approximately 10-13% of all osteoid osteomas, and poses a significant diagnostic challenge because it often lacks the typical clinical and radiographic features seen in cortical lesions [5,6]. When located in the femoral neck, patients may present with persistent hip pain, joint stiffness, synovitis, restricted range of motion, or limping, frequently leading to an initial diagnosis of inflammatory arthritis, femoroacetabular impingement, stress fracture, or osteonecrosis [5-8]. Although MRI is highly sensitive for detecting bone marrow edema and synovitis, it may miss the nidus because of extensive surrounding inflammatory changes. In contrast, CT remains the imaging modality of choice for accurately demonstrating the nidus and establishing the diagnosis, particularly in intra-articular lesions [3,6,9,10].

We report the case of a 30-year-old woman presenting with chronic right hip pain of two years’ duration, characterized by nocturnal exacerbation and partial relief with NSAIDs. Initial evaluation raised suspicion of inflammatory hip disease; however, laboratory investigations were unremarkable. Subsequent CT revealed a well-defined 10 × 8 mm nidus in the inferomedial subcapital region of the right femoral neck, establishing the diagnosis of intra-articular osteoid osteoma. The patient underwent CT-guided percutaneous excision with complete pain relief, and histopathological examination confirmed the diagnosis. To contextualize our case, we conducted a literature search of PubMed and Google Scholar through June 2026 using the keywords “osteoid osteoma,” “intra-articular osteoid osteoma,” “femoral neck,” and “hip.” We included English-language case reports, case series, and relevant review articles describing intra-articular osteoid osteoma of the hip or femoral neck. The retrieved publications were reviewed to compare the clinical presentation, imaging findings, treatment, and outcomes.

## Case presentation

A 30-year-old woman with no significant past medical history was referred to our rheumatology department for evaluation of persistent right hip pain that had progressively worsened over the preceding two years. The pain had an insidious onset, was localized to the right hip with occasional radiation to the groin, and significantly impaired daily activities, particularly walking and prolonged standing. The patient reported nocturnal exacerbation of pain with partial relief following NSAIDs. Despite repeated courses of analgesics, NSAIDs, and physiotherapy, her symptoms persisted without significant clinical improvement.

On physical examination, the patient was afebrile and in good general condition. Examination of the right hip revealed tenderness to deep palpation over the anterior hip region and mild limitation of hip motion, particularly during flexion and internal rotation. No erythema, swelling, or other local inflammatory signs were observed. Examination of the remaining joints was unremarkable. Routine laboratory investigations, including a complete blood count, C-reactive protein, and erythrocyte sedimentation rate, were within normal limits. Screening for infectious etiologies, including tuberculosis, was negative.

**Figure 1:**
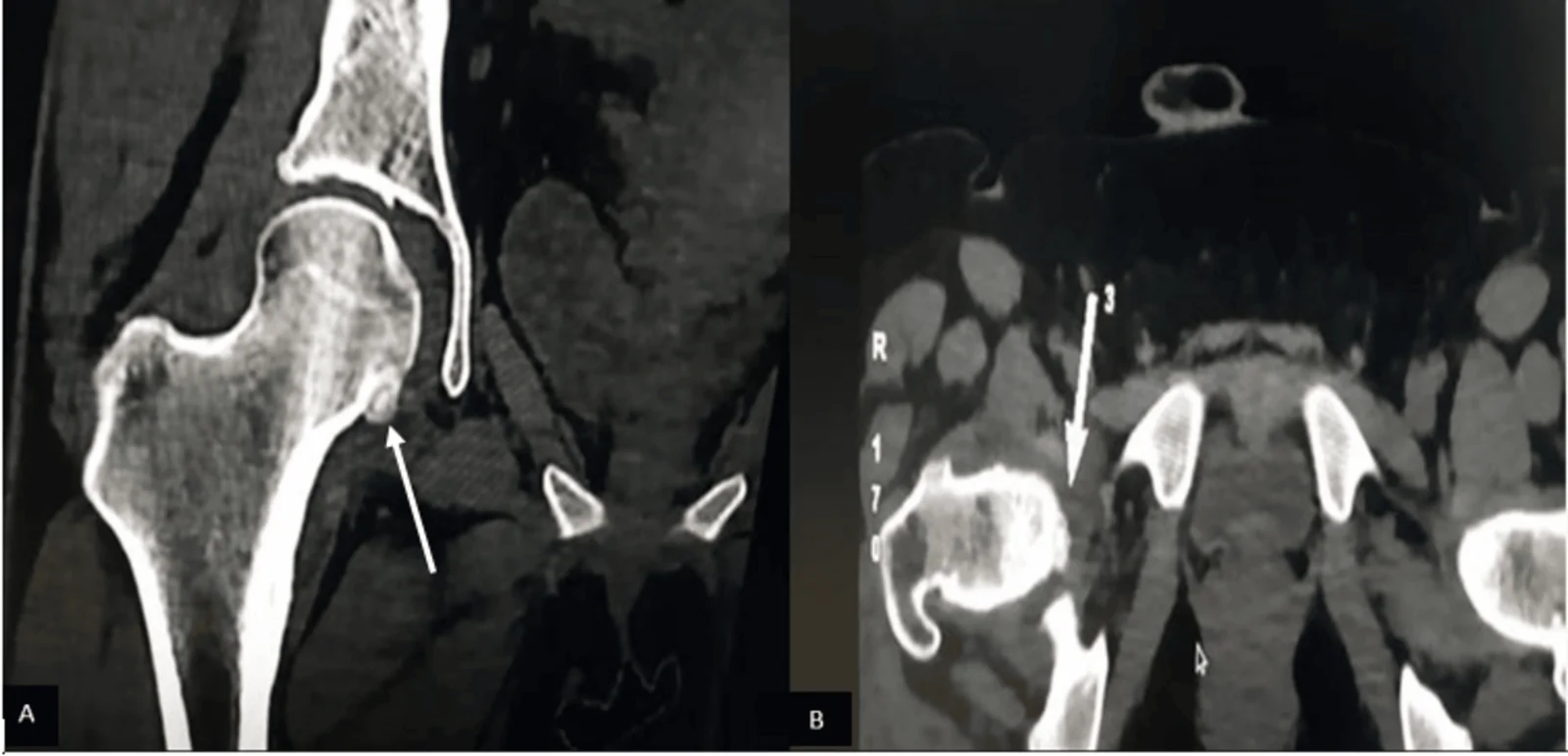
CT images of the right hip (A) Coronal CT image demonstrating a well-circumscribed nidus in the inferomedial subcapital region of the right femoral neck (arrow), surrounded by mild reactive sclerosis. (B) Axial CT image confirming the intra-articular nidus (arrow), consistent with osteoid osteoma CT: computed tomography

During the initial diagnostic work-up, inflammatory hip disease was considered due to the persistent monoarticular symptoms. Given the persistence of symptoms despite conservative treatment and the characteristic nocturnal exacerbation with partial relief after NSAIDs, further imaging with CT was performed. CT revealed a well-circumscribed 10 × 8 mm nidus in the inferomedial subcapital region of the right femoral neck, surrounded by mild reactive sclerosis, consistent with intra-articular osteoid osteoma (Figure 1).

The patient underwent CT-guided percutaneous excision of the lesion. Histopathological examination confirmed the diagnosis of osteoid osteoma. At follow-up, the patient reported complete resolution of her pain and had resumed her normal daily activities without recurrence of symptoms. For clarity, the clinical course of the patient, from symptom onset to diagnosis, treatment, and follow-up, is summarized in Table 1.

**Table 1:** Clinical timeline of the patient's diagnostic and therapeutic course NSAIDs: nonsteroidal anti-inflammatory drugs; CRP: C-reactive protein; ESR: erythrocyte sedimentation rate; CT: computed tomography

Time point	Clinical course
Two years before diagnosis	Onset of persistent right hip pain with nocturnal exacerbation and partial relief with NSAIDs
Over the following two years	Persistent symptoms despite analgesics, NSAIDs, and physiotherapy
Initial evaluation	Normal complete blood count, CRP, and ESR; infectious screening, including tuberculosis, was negative
Initial diagnostic consideration	Inflammatory hip disease was initially considered because of persistent monoarticular symptoms
Further imaging	CT revealed a well-circumscribed 10 × 8 mm nidus in the inferomedial subcapital region of the right femoral neck
Treatment	CT-guided percutaneous excision of the lesion
Histopathology	Histopathological examination confirmed osteoid osteoma
Follow-up	Complete resolution of pain and resumption of normal daily activities without recurrence of symptoms

## Discussion

Clinical presentation and diagnostic pitfalls

Osteoid osteoma classically presents with localized pain that worsens at night and is relieved by NSAIDs, a clinical pattern attributed to excessive prostaglandin production within the tumor nidus [1,3,4]. However, intra-articular lesions account for only approximately 10%-13% of all osteoid osteomas and frequently lack the characteristic clinical and radiographic features seen in cortical lesions [5,6]. When the femoral neck is involved, patients often present with persistent hip pain, restricted range of motion, synovitis, limping, or joint stiffness rather than the classic symptomatology. Consequently, these lesions are commonly misdiagnosed as inflammatory arthritis, femoroacetabular impingement, stress fracture, transient osteoporosis, or osteonecrosis of the femoral head [5-8,11].

Our patient experienced chronic hip pain for two years before the correct diagnosis was established. The chronic monoarticular presentation, despite the absence of inflammatory laboratory abnormalities, initially raised suspicion of inflammatory hip disease, illustrating one of the most common diagnostic pitfalls reported in the literature [5,7,11]. Maintaining a high index of suspicion is therefore essential, particularly in young adults with persistent monoarticular hip pain and normal inflammatory markers.

Imaging findings: the complementary roles of MRI and CT

Imaging plays a pivotal role in the diagnosis of osteoid osteoma, although its diagnostic performance varies according to the lesion location. In conventional cortical osteoid osteoma, plain radiographs often demonstrate a radiolucent nidus surrounded by reactive cortical sclerosis. However, these classic findings are frequently absent or subtle in intra-articular lesions because the periosteal reaction is limited by the intracapsular location [5,6,12].

MRI is highly sensitive for detecting reactive changes, including bone marrow edema, synovitis, joint effusion, and surrounding soft tissue inflammation. Nevertheless, these nonspecific findings may obscure the nidus and lead to misinterpretation, particularly when inflammatory arthropathy or infection is suspected [3,6,9,10,12]. Consequently, MRI alone may delay the diagnosis of intra-articular osteoid osteoma. CT is considered the most useful imaging modality for identifying the nidus because of its superior spatial resolution and ability to demonstrate the characteristic central lucency, with or without central mineralization, surrounded by variable reactive sclerosis [3,6,9,10,13].

In the present case, following the initial clinical suspicion of inflammatory hip disease, CT clearly demonstrated a well-circumscribed 10 × 8 mm nidus within the inferomedial subcapital region of the femoral neck, allowing a prompt diagnosis and appropriate management. Our findings are consistent with previous reports showing that CT is the most reliable imaging modality for diagnosing intra-articular osteoid osteoma, particularly when MRI findings are equivocal or misleading [6,9,10,12,13].

Differential diagnosis

The differential diagnosis of persistent hip pain in young adults is broad and depends on the clinical presentation, laboratory findings, and imaging features. In cases of intra-articular osteoid osteoma, the diagnosis is particularly challenging because the characteristic clinical and radiological findings may be absent [5,6,14]. Inflammatory arthritis was initially considered in our patient due to the persistent monoarticular presentation. However, the absence of elevated inflammatory markers, the chronic course of symptoms, and the characteristic nocturnal pain with partial NSAID responsiveness were not suggestive of active inflammatory arthropathy [5,6,15].

Other important differential diagnoses include femoral neck stress fracture, osteonecrosis of the femoral head, transient osteoporosis of the hip, septic arthritis, Brodie abscess, femoroacetabular impingement, and other benign bone tumors such as osteoblastoma. Careful correlation between the clinical presentation and imaging findings is essential to distinguish these entities. Unlike osteoid osteoma, stress fractures typically appear as linear cortical abnormalities, osteonecrosis is characterized by subchondral collapse or the classic "double-line" sign on MRI, and Brodie abscess is usually associated with clinical or laboratory evidence of infection [3,6,16-18].

The identification of a well-defined nidus on CT was the decisive diagnostic finding in our patient, allowing the exclusion of these alternative diagnoses and confirming intra-articular osteoid osteoma. This case underscores the importance of considering osteoid osteoma in the differential diagnosis of persistent monoarticular hip pain, particularly when MRI findings are nonspecific and laboratory investigations remain unremarkable [6,7,11,13]. Given the patient's age and clinical presentation mimicking hip synovitis, axial spondyloarthritis with hip involvement was also considered. However, the absence of inflammatory back pain, normal inflammatory markers, and the identification of a characteristic CT nidus argued strongly against this diagnosis.

Pathophysiology

The characteristic pain associated with osteoid osteoma is primarily attributed to the high concentration of prostaglandins, particularly prostaglandin E2 and prostacyclin, produced by osteoblasts within the nidus. These inflammatory mediators stimulate local nerve endings and induce marked vasodilation, thereby explaining the intense nocturnal pain and the rapid symptomatic response to NSAIDs, which inhibit cyclooxygenase activity and prostaglandin synthesis [1,3,4,19].

In intra-articular lesions, the close proximity of the nidus to the synovium results in reactive synovitis, joint effusion, capsular thickening, and bone marrow edema. These secondary inflammatory changes may mask the underlying lesion on MRI and alter the classic clinical presentation, often leading to misdiagnosis as inflammatory arthritis or other inflammatory joint disorders [5,6,9,10,20]. A thorough understanding of these pathophysiological mechanisms is essential for interpreting atypical imaging findings and facilitating early recognition of intra-articular osteoid osteoma during the diagnostic process [3,5,6].

Therapeutic management

The treatment of osteoid osteoma has evolved from open surgical excision to minimally invasive image-guided techniques, which are now considered the standard of care for most lesions because of their high success rates, low morbidity, and rapid postoperative recovery [3,21-24]. Currently, CT-guided percutaneous procedures are widely accepted as the preferred treatment for most osteoid osteomas because they allow precise localization of the nidus while minimizing damage to the surrounding healthy bone [21-24]. Among these techniques, CT-guided radiofrequency ablation (RFA) is the most widely used, with reported clinical success rates exceeding 90%. Nevertheless, CT-guided percutaneous excision remains an excellent alternative when histopathological confirmation is required or when tissue sampling is clinically indicated [21-25].

In the present case, CT-guided percutaneous excision enabled removal of the lesion with subsequent histopathological confirmation of osteoid osteoma, resulting in rapid resolution of symptoms without complications. This approach provided both definitive diagnosis and treatment in a single procedure. Treatment should be individualized according to lesion location, available expertise, and the need for histopathological confirmation. In our patient, CT-guided percutaneous excision provided both definitive treatment and histological confirmation, illustrating the effectiveness of this approach in selected intra-articular lesions [21-25].

Comparison with the published literature

To better contextualize our findings, we compared the present case with previously reported cases of intra-articular and femoral neck osteoid osteoma (Table 2). Although patient characteristics, lesion location, and treatment strategies varied across studies, several consistent clinical and radiological patterns emerged.

**Table 2:** Comparison of the present case with previously reported cases of intra-articular or femoral neck osteoid osteoma MRI: magnetic resonance imaging; CT: computed tomography

Study	Initial diagnosis	Key imaging findings	Treatment	Clinical lesson
Present case	Inflammatory hip disease	CT demonstrated a 10 × 8 mm nidus in the inferomedial subcapital femoral neck	CT-guided percutaneous excision with histopathological confirmation	Osteoid osteoma should be suspected in patients with persistent monoarticular hip pain despite normal inflammatory markers. CT is essential when initial clinical evaluation or preliminary imaging is inconclusive
Allen and Saifuddin [6]	Various inflammatory and degenerative hip disorders	MRI frequently demonstrated bone marrow edema and synovitis, whereas CT clearly identified the nidus	Imaging review	MRI may be misleading in intra-articular osteoid osteoma; CT remains the imaging modality of choice
Lee et al. [11]	Delayed or incorrect diagnosis	CT accurately demonstrated the nidus after inconclusive initial imaging	Surgical excision	Early recognition of the characteristic clinical presentation reduces diagnostic delay
Kale and Chowdhary [26]	Avascular necrosis of the femoral head	CT demonstrated a femoral neck nidus after misleading MRI findings	Surgical excision	Osteoid osteoma should be considered in the differential diagnosis of persistent hip pain mimicking avascular necrosis
Gillet et al. [27]	Various hip disorders	CT was the cornerstone for diagnosis and image-guided management	Image-guided interventional treatment	Modern CT-based imaging and minimally invasive treatment improve diagnostic accuracy and clinical outcomes
Desi et al. [28]	Atypical intra-articular presentation	CT accurately localized the nidus before ablation	CT-guided radiofrequency ablation	CT-guided ablation is safe and effective even in challenging anatomical locations
Lao et al. [29]	Persistent hip pain	CT identified the femoral neck nidus before robot-assisted excision	Robot-assisted endoscopic excision	Accurate CT localization allows successful minimally invasive surgery with excellent outcomes

As summarized in Table 2, intra-articular and femoral neck osteoid osteoma are consistently associated with an atypical clinical presentation, frequent diagnostic delay, and misleading MRI findings [5-13]. In most published reports, patients initially received alternative diagnoses, including inflammatory arthritis, femoroacetabular impingement, stress fracture, or other hip disorders, reflecting the nonspecific manifestations of this uncommon entity [7,8,11,26,27].

Several limitations should be considered when interpreting the published cases summarized in Table 2. Case reports and case series are inherently subject to publication and selection bias, and the available literature is heterogeneous with respect to clinical presentation, lesion location, imaging modalities, and treatment approaches. Therefore, these reports cannot establish comparative diagnostic accuracy or therapeutic effectiveness. The purpose of the present comparison is primarily to illustrate recurring diagnostic patterns and management strategies reported in patients with intra-articular or femoral neck osteoid osteoma.

Across the available literature, CT has consistently proven to be the most accurate imaging modality for identifying the nidus and establishing the diagnosis, particularly when conventional radiographs or MRI are inconclusive [6,9,10,12,26]. Likewise, minimally invasive CT-guided procedures, especially radiofrequency ablation and percutaneous excision, have demonstrated excellent clinical outcomes with low complication rates, whereas open surgical excision remains an effective alternative in selected cases [21-25,27].

Our patient closely paralleled these previously reported cases, presenting with prolonged hip pain and a two-year diagnostic delay before CT demonstrated the characteristic femoral neck nidus. The successful outcome following CT-guided percutaneous excision with histopathological confirmation further supports the pivotal role of CT not only in establishing the diagnosis but also in facilitating definitive treatment. Collectively, these findings reinforce the importance of maintaining a high index of suspicion for osteoid osteoma in young adults presenting with persistent monoarticular hip pain despite normal inflammatory markers.

Clinical implications

From a clinical perspective, intra-articular osteoid osteoma should be considered in the differential diagnosis of persistent monoarticular hip pain in young adults, particularly when symptoms persist despite conservative management and inflammatory markers remain normal. The combination of nocturnal pain and partial relief with NSAIDs should raise clinical suspicion, even when reactive changes suggestive of inflammatory hip disease are initially suspected during preliminary evaluation. Although MRI is highly sensitive for detecting bone marrow edema and joint effusion, the literature demonstrates that it may fail to identify the tumor nidus; therefore, CT should be performed early whenever osteoid osteoma is suspected clinically. Timely diagnosis via CT enables minimally invasive targeted treatment, provides rapid symptomatic relief, and avoids unnecessary diagnostic delays, inappropriate therapeutic interventions, and prolonged patient morbidity.

## Conclusions

Intra-articular osteoid osteoma of the femoral neck is an uncommon but important cause of persistent hip pain in young adults and should be considered in patients with chronic monoarticular hip pain, particularly when inflammatory markers are normal and symptoms persist despite conventional treatment. Its atypical clinical presentation and nonspecific MRI findings may contribute to diagnostic delay. This case highlights the value of CT in identifying the characteristic nidus when MRI findings are inconclusive or misleading. Prompt recognition of this entity may help reduce diagnostic delay, unnecessary investigations, and inappropriate therapeutic interventions. In our patient, CT-guided percutaneous excision resulted in complete pain relief and provided histopathological confirmation of the diagnosis. The published cases reviewed in this report should be interpreted cautiously given their heterogeneous nature and potential publication and selection bias.
